# 

**Published:** 1888-08

**Authors:** 


### BOOK NOTICES.

THE HEALTH COURT, Seatle, Washington Territory. Numbers 1 and 2 of this new quarterly, published by Dr. A. Devou at 25 cents per year. An exceedingly instructive sheet upon all matters pertaining to Hygiene, and worth double the money.

PETER COOPER.—Take him all in all, Peter Cooper was the greatest Philanthropist New York has ever had. His monument is to be seen in the Cooper Union, the noblest testimonial which any New Yorker has left behind him for the advancement of the generations that follow.

The catalogue of the current year, giving a list of the large number of pupils in the various Scientific and Art Schools, attests the great value of these free departments to the city and to those who are so fortunate to avail themselves of their privileges.

OPERATIVE SURGERY.—We are indebted to Prof. Donald Maclean, M.D. of Detroit, for valuable treatise on Operative Surgery; to Prof. Henry J. Reynolds, M. D. of Chicago, for a pamphlet on the uses of Cocaine Anæsthesis in delicate surgical operations; to A. E. Prince, M. D. of the Wabash, Ind. Deaf, Dumb and Blind Institution, for valuable hints on the treatment of granulated Eyelids; to H. F. Hendrix, M. D., for reprint of his remarks on Cholera Infantum and its treatment read before the St. Louis Medical Society, June 2, to whom severally our thanks are due.

THE HORTICULTURAL ART JOURNAL, of Rochester, N. Y., for July, contains among other things, some beautifully colored lithographs of Apples and much valuable information upon kindred subjects.

THE CENTURY.—The Century still holds the lead among our enterprising monthlies, its Lincoln history, Siberian papers, war sketches, and pictures of ranch life, with their illustrations are continued with unflaging interest.

The great fire which lately demolished a portion of its printing establishment and much valuable property, has not interfered with its regular monthly issue, or in anywise depressed its energies. Address, Century Company, N. Y. City.

